# Psychopathological symptoms associated with synthetic cannabinoid use: a comparison with natural cannabis

**DOI:** 10.1007/s00213-019-05238-8

**Published:** 2019-04-09

**Authors:** Vincent T. Mensen, Annabel Vreeker, Johan Nordgren, Amanda Atkinson, Rafael de la Torre, Magi Farré, Johannes G. Ramaekers, Tibor M. Brunt

**Affiliations:** 1 Department of Drug Monitoring, Utrecht, The Netherlands; 2 University Medical Center Utrecht, Department of Psychiatry, University of Utrecht, Utrecht, The Netherlands; 3 Department of Social Work, Malmö University, Malmö, Sweden; 4 Public Health Institute, Liverpool John Moores University, Liverpool, UK; 5 Integrative Pharmacology and Systems Neurosciences, Hospital del Mar Medical Research Institute (IMIM), Barcelona, Spain; 6 Clinical Pharmacology, Hospital Universitari Germans Trias i Pujol, Badalona, Universitat Autònoma de Barcelona, Bellaterra, Spain; 7 Department of Neuropsychology and Psychopharmacology, Faculty of Psychology and Neuroscience, Maastricht University, Maastricht, The Netherlands; 8 Department of Psychiatry, Academic Medical Centre, University of Amsterdam, Meibergdreef 9, Amsterdam, The Netherlands; 9 Department of Developmental Psychopathology, Radboud University, Nijmegen, The Netherlands

**Keywords:** Cannabis, Synthetic cannabis, Spice, Mental health, Psychology, Psychiatry, Questionnaire, BSI, ISI, Altman, DUDIT

## Abstract

**Background:**

Synthetic cannabinoids (SCs) are a class of new psychoactive substances that have been rapidly evolving around the world throughout recent years. Many different synthetic cannabinoid analogues are on the consumer market and sold under misleading names, like “spice” or “incense.” A limited number of studies have reported serious health effects associated with SC use. In this study, we compared clinical and subclinical psychopathological symptoms associated with SC use and natural cannabis (NC) use.

**Methods:**

A convenience sample of 367 NC and SC users was recruited online, including four validated psychometric questionnaires: The Drug Use Disorders Identification Test (DUDIT), Insomnia Severity Index (ISI), Altman Mania Scale (Altman), and Brief Symptom Inventory (BSI). The two groups were compared with analysis of variance (ANOVA) and covariance (ANCOVA), chi^2^ tests, and logistic regression when appropriate.

**Results:**

The SC user group did not differ in age from the NC user group (27.7 years), but contained less females (21% and 30%, respectively). SC users scored higher than NC users on all used psychometric measures, indicating a higher likelihood of drug abuse, sleep problems, (hypo)manic symptoms, and the nine dimensions comprising the BSI, somatization, obsessive-compulsive behavior, interpersonal sensitivity, depression, anxiety, hostility, phobic anxiety, paranoid ideation, and psychoticism. Odds ratios (95% CI) for the SC user group vs NC user group were, respectively, drug dependence 3.56 (1.77–7.16), (severe) insomnia 5.01 (2.10–11.92), (hypo-)mania 5.18 (2.04–13.14), and BSI psychopathology 5.21 (2.96–9.17).

**Discussion:**

This study shows that SC use is associated with increased mental health symptomatology compared to NC use.

## Introduction

There has been a dramatic increase in the diversity of new psychoactive substances (NPS) that are being sold as recreational drugs on the consumer market, mostly through the internet (European Monitoring Centre for Drugs and Drug Addiction 2015, 2017). The number of different NPS that has been reported to the early warning system of the European Union is already up to 600 since 2005 and is still increasing. Whereas the diffusion of NPS on the consumer market initially started out with stimulants, such as synthetic cathinones (Bossong et al. 2005; Brunt et al. 2011), these were followed by a wide array of other chemicals belonging to a range of drug classes. These include hallucinogens such as n-benzyl-oxy-methyl derivatives (NBOMes) and the 2C-class drugs (Burns et al. 2014; Caudevilla-Gálligo et al. 2012; Hondebrink et al. 2015), entactogens such as benzofurans (Hondebrink et al. 2015; Soh and Elliott 2014) and synthetic opioids and the cannabis analogues, synthetic cannabinoids (SCs) (Martinotti et al. 2017).

SCs refer to herbal or powder preparations containing synthetic cannabinoid receptor agonists. SCs were originally developed in order to investigate the selective therapeutic effects of Δ9-tetrahydrocannabinol (THC) and the role of the endocannabinoid system by selectively targeting cannabinoid receptors (Pertwee 2006; Huffman et al. 1996). In fact, some potential for therapeutic purposes was found for certain synthetic cannabinoid agonists and antagonists (De Luca and Fattore 2018; Muller et al. 2019). The SCs currently used recreationally are potent cannabinoid receptor agonists often synthesized in clandestine laboratories in China with a much higher receptor affinity and more intense psychoactive effects than THC, which is a partial agonist of these receptors (Castaneto et al. 2014; ElSohly et al. 2014; European Monitoring Centre for Drugs and Drug Addiction 2017; Hoffman et al. 2017; Schifano et al. 2015; van Amsterdam et al. 2015).

SCs form the largest category of NPS: they represent 32% of all NPS and a total of over 240 different SCs have been reported to the United Nations Office on Drugs and Crime by 65 member states (United Nations Office on Drugs and Crime 2017). In addition, the quantity of SCs seized between 2010 and 2015 showed a sharp increase. SCs are usually sold in colorful packaging marked with labels to suggest that it is not for human consumption, such as “incense” or “spice” in order to mislead and circumvent drug laws. Different countries have different laws concerning SCs, sometimes via compound-specific bans or via generic cannabinoid analogue legislation by a blanket ban. SCs are still easily obtained however, especially via the internet (European Monitoring Centre for Drugs and Drug Addiction 2015, 2017). SCs are mainly used out of curiosity, to avoid detection in routine drug tests, and because of availability and non-illegality (Vandrey et al. 2012; Winstock and Barratt 2013).

The relatively high potency compared to natural cannabis and increasing prevalence of use of SCs together with an unfamiliarity with their effects have led to an increase in the number of cases with acute SC intoxication around the world. In Russia, Poland, and the USA, SCs have been associated with poisoning and deaths (Adamowicz 2016; Law et al. 2015; Shevyrin et al. 2015). Systematic data on prevalence is scarce, partly because SCs do not show up in most standard toxicological screens in saliva or urine. In the USA, the number of emergency department visits related to SCs increased from 11,406 to 28,531 between 2010 and 2011 (Bush and Woodwell 2013). In Europe, SCs were reported in 0.3% of all drug toxicity cases in 16 emergency rooms (Dines et al. 2015). Most patients present with relatively mild physical (nausea, shortness of breath, hypertension) and mental (restlessness, agitation, and anxiety) symptoms and recover within 24 h with supportive care. In some cases, however, patients are hospitalized for serious physical (myocardial infarction, stroke, seizures, kidney injury) and mental (psychosis, paranoia) complications associated with SC use (Castaneto et al. 2014; Musshoff et al. 2014; Tait et al. 2016). While causality is difficult to establish, and many SC users also use other drugs (Winstock and Barratt 2013), case studies suggested that SC use is able to trigger long-term psychotic symptoms in otherwise healthy individuals (Fattore 2016). Furthermore, among psychiatric patients, SC users presented with more severe psychotic symptoms and agitation compared to natural cannabis (NC) users (Bassir Nia et al. 2016; Shalit et al. 2016).

While this data from emergency rooms and clinics highlights the risks associated with SCs, little is known about the majority of recreational users, not admitted in any health care system. A couple of studies have appeared recently, indicating a higher proneness to abuse or risk-taking behavior in adolescent users of SC compared to users of natural cannabis (Clayton et al. 2017; Blevins et al. 2016). From online discussions we know that recreational users report acute symptoms very similar to those observed in emergency rooms, both physically (tachycardia, respiratory issues, nausea) and mentally (fear, memory impairment), in addition to sub-acute (hangovers, lethargy) and longer-term issues (dependence, memory impairment, mood swings) (Soussan and Kjellgren 2014). This study aims to quantify a wide range of psychopathological symptoms in a group of recreational SC users in a non-clinical setting and compare them to natural cannabis (NC) users.

## Methods

### Sample

This convenience sample included SC and NC users, recruited in three European countries (United Kingdom, Sweden, and the Netherlands). The subjects have been contacted through social media (Facebook, Twitter) and by drug-related forums that are the most used sales/diffusion channels, as reported by a review (Miliano et al. 2018). The online drug-related forums included www.flashback.org and www.bluelight.org, from January to June 2017. As prevalence of SC use is rather uncertain, we directed most effort into recruiting users of SC through the various nightlife and drug-related forums and social media.

Inclusion criteria for the participants were that they had used either natural cannabis and/or synthetic cannabinoids in the past 12 months and were 18 years of age or older. Participants were obligated to give online informed consent before filling out the survey and time to complete the survey took approximately 25 min. The study was approved by the research ethical committee of Maastricht University.

### Survey and psychometric measures

First of all, the survey started with some demographic items, like age, gender, education, work status, and housing status. The next part dealt with substance use history, in which last year use and past use (last 2 weeks) was asked for several substances, including alcohol and tobacco.

To get a broad overview of any potential psychological problems, four standardized psychometric instruments were used, based on symptoms that were frequently mentioned in relationship to (synthetic) cannabinoids in scientific literature (e.g., depression, psychosis, anxiety, sleep disorders, abuse, and dependence):

To assess the level of substance abuse problems in both groups, we used the 11-item Drug Use Disorders Identification Test (DUDIT) (Berman et al. 2005; Hildebrand 2015). The DUDIT consists of nine questions on a 5-point scale, and two questions on a 3-point scale, all of which contribute to a total score which can fall in three categories: no problems, substance abuse (6+), or substance dependence (20+). To assess any problems surrounding sleep disturbances and insomnia, the Insomnia Severity Index (ISI) was used (Morin et al. 2011). This is a 7-item questionnaire where the participants rate sleep-related problems on a 5-point scale which all contribute to a total score which can be categorized as no problems, subthreshold insomnia (8+), clinical insomnia (15+), and severe clinical insomnia (22+). The Altman Self-Rating Mania Scale (Altman) contains 5 questions which are answered on a 5-point scale and is used to assess the presence and severity of (hypo)manic symptoms (Altman et al. 2001; Altman et al. 1997). The total score on these 5 questions can be categorized as no symptoms, or a high probability of a (hypo)manic condition (6+). To gain insight into any other mental problems, the Brief Symptom Inventory (BSI) was used (Derogatis 1993). It contains 53 questions which are answered on a 5-point Likert scale. Each question contributes to a global severity index as well as one of nine psychopathological dimensions: somatization, obsessive-compulsive, interpersonal sensitivity, depression, anxiety, hostility, phobic anxiety, paranoid ideation, and psychoticism.

### Statistics

First, we compared the SC and NC user groups to each other on age using analysis of variance (ANOVA) and gender using a chi^2^ test. For our main analyses, we compared the SC users to the NC users. We compared the SC and NC user groups on age using ANOVA, and gender, frequency of use, and use of other drugs using chi^2^ tests. We then compared both groups on the primary outcome measures (DUDIT total score, ISI total score, Altman total score, and the BSI global severity index, or GSI) using ANCOVA with the primary outcome measure as dependent variable, group (SC vs NC) as independent variable, and age and gender as covariates. The nine categories in the BSI were then analyzed separately using this same method.

#### Clinical outcome analysis

To analyze the relative risk of clinically relevant mental health problems of SC use compared to NC use we investigated the categorical outcome measures of the questionnaires.

The DUDIT has three outcome categories related to the total score: no problems (< 6), drug abuse (6–19), and drug dependence (20+). We compared the no problem group to the other two groups.

The ISI has four outcome categories related to the total score: no significant insomnia (< 8), subthreshold insomnia (8–14), clinical insomnia (15–21), and severe clinical insomnia (22+). We compared the participants with clinical and severe clinical insomnia groups to the participants with no or subthreshold insomnia.

The Altman mania scale has two outcome categories: no manic symptoms (< 6) or (hypo)manic symptoms (6+). These outcome categories were compared.

The BSI judges an individual to be a potential psychiatric case with a GSI score equal to or greater than a T-score of 63. For adult non-patients this translates to a GSI score of 0.58 for men and 0.78 for women according to Appendix A of the BSI manual (Derogatis 1993). Participants were assigned to two groups (psychiatric problems or not) accordingly.

These categories were analyzed using logistic regression, with user group (SC or NC) as independent variable, clinical category as dependent variable, and age and gender as covariates. Bias-corrected accelerated bootstrapping was used to produce confidence intervals.

## Results

### Participants

The total number of responses was 589 SC users and 417 NC users. From this total, cases were removed if they did not give explicit consent, if they did not provide an age over 18 or gender, if they did not complete at least one of the questionnaires, and if they did not explicitly state they had used NC (for the NC users) or SC (for the SC users) in the past year. After removing these cases, we included 367 participants from three different countries: 238 SC users and 129 NC users (see Table 1). For all analyses, cases with missing data on any measure were pairwise excluded.

**Table 1:** Participant information

Substance	Age range (years old)	Mean age (years old ± SD)	Women	Men	Other
SC users (*N* = 238)	18–66	27.6 (± 8.5)	51 (21%)	177 (74%)	10 (4%)
NC users (*N* = 129)	18–71	27.7 (± 10.2)	39 (30%)	90 (70%)	0 (0%)

SD standard deviation, SC synthetic cannabis, NC natural cannabis

### Age and gender

There was no difference in age between the SC and NC user groups (*F*(1,365) = 0.575, *p* = 0.449). There were fewer women in the SC user group (21%) than in the NC user group (30%) (chi^2^ = 8.308, *p* = 0.016).

### Substance use

There was a difference in frequency of use (chi^2^ = 100.029, *p* < 0.001), with significantly fewer SC users (30%) reporting monthly or more frequent SC use than the NC use among the NC users group (84%). There was no difference in duration of use (as calculated by the intervals for age of onset and current age of SC use and NC use) between the NC user group and the SC user group.

Use of 12 psychoactive drugs in the previous year and past 2 weeks was assessed. NC use was also highly prevalent in the SC user group; SC use was non-prevalent in the NC user group however. NC use was lower in the SC user group only in the last 2 weeks of reported substance use. In addition, salvia was used by more SC users in the past 2 weeks and past 12 months (8.5%) than NC users (0%). There was no difference between groups for all other substances at a Bonferroni corrected *p* value of 0.004 (see Tables 2 and 3).

**Table 2:** Additional psychoactive drug use in past 12 months

Drug	NC user group	SC user group	Chi^2^
Natural cannabis	100.0%	81.5%	2.561, *p* = 0.126
Amphetamines	18.7%	31.3%	3.871, *p* = 0.051
Cocaine/crack	9.0%	15.4%	2.888, *p* = 0.115
Tranquilizers	9.3%	19.2%	4.925, *p* = 0.024
Ecstasy/MDMA	5.4%	9.3%	2.056, *p* = 0.189
LSD/other hallucinogens	4.8%	7.5%	3.974, *p* = 0.067
Magic mushrooms	0.7%	4.2%	4.041, *p* = 0.087
GHB/GBL	0.0%	1.9%	4.122, *p* = 0.065
Ketamine	2.0%	8.1%	6.877, *p* = 0.106
Amyl nitrate/poppers	4.1%	1.4%	0.711, *p* = 0.612
*Salvia divinorum*	0.0%	8.8%	9.129, *p* = 0.000*
Nitrous oxide/laughing gas	4.1%	5.6%	0.874, *p* = 0.471
Methylone	4.6%	3.8%	2.125, *p* = 0.231

SC synthetic cannabis, NC natural cannabis

*Significant at Bonferroni-corrected *p* = 0.004

**Table 3:** Additional psychoactive drug use in the past 2 weeks

Drug	NC user group	SC user group	Chi^2^
Natural cannabis	74.7%	21.3%	7.862, *p* = 0.002*
Amphetamines	11.0%	21.5%	3.594, *p* = 0.071
Cocaine/crack	6.1%	12.3%	2.419, *p* = 0.155
Tranquilizers	7.1%	17.1%	4.634, *p* = 0.044
Ecstasy/MDMA	3.4%	7.3%	1.735, *p* = 0.212
LSD/other hallucinogens	2.1%	6.9%	4.865, *p* = 0.033
Magic mushrooms	0.0%	3.8%	4.512, *p* = 0.046
GHB/GBL	0.0%	1.4%	3.855, *p* = 0.085
Ketamine	1.2%	7.3%	6.138, *p* = 0.120
Amyl nitrate/poppers	4.1%	1.4%	0.560, *p* = 0.517
*Salvia divinorum*	0.0%	8.4%	9.929, *p* = 0.000*
Nitrous oxide/laughing gas	3.4%	4.9%	0.982, *p* = 0.381
Methylone	3.6%	1.6%	6.950, *p* = 0.013

SC synthetic cannabis, NC natural cannabis

*Significant at Bonferroni-corrected *p* = 0.004

### Psychometric instruments

The SC user group showed a higher DUDIT total score than the NC user group (*F*(1,357) = 21.336, *p* = 0.000, eta^2^ = 0.056). Adjusted means: SC user group 13.73 (95% CI 12.49–14.96), NC user group 9.36 (95% CI 8.30–10.40). Likewise, the SC user group showed a higher ISI total score than the NC user group (*F*(1,222) = 20.052, *p* < 0.001, eta^2^ = 0.083). Adjusted means: SC 9.66 (95% CI 8.50–10.83), NC 5.60 (95% CI 4.61–6.70) (Table 4).

**Table 4:** Means adjusted for gender and age, between-subject significance, and partial eta squared for the effect of group on the questionnaires

Questionnaire	NC adjusted mean	SC adjusted mean	*p*	Partial eta^2^
DUDIT	9.36	13.73	< 0.001	0.056
ISI	5.60	9.66	< 0.001	0.083
Altman	1.72	3.84	< 0.001	0.095
BSI (GSI)	0.38	1.00	< 0.001	0.147

DUDIT Drug Use Disorders Identification Test, ISI Insomnia Severity Index, BSI Brief Symptom Inventory, GSI General Symptom Inventory, SC synthetic cannabis, NC natural cannabis

The Altman total score was higher in the SC user group than the NC user group (*F*(1,229) = 23.982, *p* < 0.001, eta^2^ = 0.095). Adjusted means: SC 3.84 (95% CI 3.23–4.43), NC 1.72 (95% CI 1.27–2.14). The SC user group showed a higher GSI total score compared to the NC user group (*F*(1,289) = 49.936, *p* = 0.000, eta^2^ = 0.147). Adjusted means: SC 1.00 (95% CI 0.87–1.14), NC 0.38 (95% CI 0.31–0.44). This was the case for all nine BSI categories underlying the GSI (Table 5).

**Table 5:** The 95% confidence intervals adjusted for gender and age, between-subject significance, and partial eta squared for the effect of group on BSI category score

BSI category	NC 95% CI	SC 95% CI	*p*	Partial eta^2^
Somatization	0.23–0.36	0.61–0.87	< 0.001	0.088
Obsessive-compulsive	0.49–0.72	1.20–1.53	< 0.001	0.131
Interpersonal sensitivity	0.30–0.49	0.77–1.10	< 0.001	0.078
Depression	0.43–0.70	1.05–1.39	< 0.001	0.088
Anxiety	0.24–0.42	1.00–1.32	< 0.001	0.173
Hostility	0.15–0.28	0.58–0.86	< 0.001	0.089
Phobic anxiety	0.19–0.36	0.77–1.10	< 0.001	0.115
Paranoid ideation	0.17–0.28	0.75–1.05	< 0.001	0.149
Psychoticism	0.27–0.45	0.67–0.98	< 0.001	0.068

BSI Brief Symptom Inventory, CI confidence interval, SC synthetic cannabis, NC natural cannabis

### Clinical outcome analysis

Using logistic regression to examine the clinical outcome measures of the psychometric instruments, we found that participants in the SC user group were between 3.56 and 5.21 times more likely than participants in the NC user group to score in the clinical range of the psychometric instruments (Table 6).

**Table 6:** The relationship between clinical symptoms in the SC group compared to the NC group

Clinical measure	Questionnaire	OR SC vs NC	95% CI	*p*
Drug dependence	DUDIT	3.56	1.77–7.16	< 0.001
(Severe) insomnia	ISI	5.01	2.10–11.92	< 0.001
(Hypo-)mania	Altman	5.18	2.04–13.14	0.001
Psychiatric problems	BSI	5.21	2.96–9.17	< 0.001

Logistic regression with age and gender as covariates

OR odds ratio, CI confidence interval, DUDIT Drug Use Disorders Identification Test, ISI Insomnia Severity Index, BSI Brief Symptom Inventory

## Discussion

In this study we found that compared to NC use, SC use is more strongly associated with a broad range of self-reported mental health problems. The SC user group scored significantly higher on all measured indices of general and specific psychopathology, and odds of scoring in the clinical range on any of our measurements were 3.5 to over 5 times higher in the SC user group compared to the NC user group. These results warrant serious concern about SC use.

Few studies have attempted to investigate mental health problems associated with SC use in a non-clinical population. In a clinical population differences between NC and SC users were observed, indicating an association of SC use with psychotic problems, agitation, and longer hospitalizations (Bassir Nia et al. 2016; Shalit et al. 2016). While the studied populations are very different, this supports our findings of more severe problems related to SC use compared to NC use. On our main index of psychiatric problems, the BSI, SC users have higher scores than NC users on the GSI and all underlying categories. The probability (odds ratio) of the GSI to exceed the threshold for a psychiatric diagnosis, as is formulated in the BSI manual (Derogatis 1993), is five times higher for participants in the SC group than the NC group. This is especially worrying considering that many studies have found that NC use itself is already more often associated with psychiatric problems, including depression, anxiety, and in particular psychotic symptoms and schizophrenia (Moore et al. 2007; Volkow et al. 2014).

Aside from the general psychiatric problems indexed by the BSI, the results from the other psychometric measures were also in line with previous studies. Cannabis use has been associated with insomnia symptoms and impairment of sleep quality in various studies (Babson et al. 2017; P. Gates et al. 2016; P. J. Gates et al. 2014). Mainly, long-term use of cannabis (mainly the chief component THC) seems to have a negative impact on sleep quality, whereas some cannabinoids show some promise for treatment of insomnia (Babson et al. 2017). However, the causative relationship of cannabis and insomnia remains unclear and insomnia might as well be a risk factor for adolescent cannabis use (Roane and Taylor 2008). Notwithstanding cause and effect, our study confirms a relationship between insomnia symptoms and the use of cannabinoid receptor agonists, whereby use of SCs is associated with poorer sleep quality than use of NC.

Cannabis use has also been associated with increased risk of mania and subsequent bipolar disorder (Strakowski et al. 2007). Individuals with bipolar disorder were 6.8 times more likely to report a lifetime history of cannabis use (Agrawal et al. 2011). Cannabis use may also cause incidental episodes of mania or even long-term bipolar disorder, with cannabis use often preceding onset of the first mania (Bally et al. 2014; Henquet et al. 2006). Furthermore, NC use was also associated with a younger age of onset of the first mania and an exacerbation of depressive and manic symptomatology in patients diagnosed with bipolar disorder (Bally et al. 2014; Gibbs et al. 2015). The results of our study underline this association between the cannabinoid system and mania, while demonstrating that SCs show a greater association with mania or bipolar disorder.

NC use may also lead to substance use disorders or dependence (Hall and Degenhardt 2009). The results of our study suggest a much higher dependence liability of SCs than NC. There is evidence to suggest that SCs, with a much greater potency and affinity for the endocannabinoid system, are capable of inducing dependence. For instance, recent neuroimaging research showed a modest, but significant increase of striatal dopamine in humans after cannabis use and also impaired striatal release in dependent cannabis users (Bossong et al. 2009; van de Giessen et al. 2017). Our findings of a higher DUDIT score for SC users are supported by a study executed among high school students, showing higher substance use disorder psychopathology (Blevins et al. 2016). The higher DUDIT score in the SC user group cannot be attributed to frequency of use, which was higher for NC in the NC user group. However, it has to be stipulated that the DUDIT is not able to differentiate between which specific substance is most likely to be underlying increased DUDIT scores and most users are poly-drug users.

Our results on SCs are also supported by in vitro and animal studies at the moment. The pharmacological effects are similar to the main active ingredient of NC: Δ9-THC, but with a much greater binding affinity for the cannabinoid receptors, in particular the cannabinoid 1 receptor (CB1R) (Castaneto et al. 2014; Martinotti et al. 2017). This role as a potent CB1R agonist has been observed to cause disturbed synaptic functioning, potentially much stronger in SCs than Δ9-THC which is a partial agonist (Hoffman et al. 2017). Furthermore, SC mixtures or SCs alone do not contain cannabidiol, a compound found in NC that is associated with reducing psychotic experiences and anxiety, further increasing the potential risk of mental health problems emerging after the use of SCs (Di Forti et al. 2015; Murray et al. 2016; van Amsterdam et al. 2015). Even though there is much debate on the causality of NC use and psychiatric problems, potent activation of CB1 receptors by increasingly potent agonists is a likely mechanism to cause long-term brain changes (Haney and Evins 2016). Combining our findings of much higher scores on all indices of psychiatric problems with the knowledge from in vitro and animal studies about SC potency, again strong caution is warranted in the case of SC use.

While our data clearly reveal that SC use is associated with increased mental health problems, there are some limitations to the study. First of all, it is a study which relies on effects and symptoms that are self-reported on recall basis. And, because it is a cross-sectional study, no statements can be made regarding causality as we do not know if SC leads to increased mental health problems, or if mental health problems instigate SC use. To investigate causality, future studies should employ a longitudinal design. In addition, the fact that the majority of SC users also used NC in the past year might substantially confound current findings, since effects of NC could be underlying effects associated with the SC using group. Still, it does not confound the magnitude in difference in symptoms found between the two groups. There were no general statistical differences found in most other substances used between these two groups. However, we cannot discount the role other drugs in the development of mental health status of our participants. Furthermore, recruitment methods might underlie some differences we found, as different drug-related forums and social media were used to suit each country, which might have resulted in bias. However, they were selected to optimize sample size, and using identical recruitment platforms, if even possible, would have likely led to small sample sizes with its own limitations. Since this study is based on data acquired using an online survey, the veracity of the answers cannot be determined. Notwithstanding this argument, online surveys do not appear to differ from traditional surveys in this regard (Miller et al. 2002). Finally, we have no information on the subtype or amount of SC used by the participants. Unfortunately, this mimics the real-world situation and highlights an additional danger of SC use. There is significant variance of subtype and quantity of SCs, even between packages with the same name (Fattore 2016).

Our study demonstrates that there are significant mental health problems in this population of SC users, which should be cause for concern for treatment and prevention professionals. Use of SCs is associated with a probability for mental health risks up to over five times greater than NC use, which is in line with previous results assembled in clinical and preclinical (experimental animal) studies with SCs which showed a much more potent mechanism of action than NC. This study should be considered as a strong caution about the potential danger of SC use to mental health.
